# A serum cytokine-derived integrated inflammatory profile score (IIPS) links systemic inflammation to symptom burden and severity in COPD

**DOI:** 10.7717/peerj.21751

**Published:** 2026-10-01

**Authors:** Jiexun Cai, Zhihui Li, Yanfeng Yi, Xiangli An, Anqi Li, Hui Han, Xiaoyu Li, Yunliang Yao

**Affiliations:** 1 Deqing People’s Hospital and The Affiliated Deqing Hospital, Huzhou University, Huzhou, Zhejiang, China; 2 School of Life and Health Sciences, Huzhou College, Huzhou, Zhejiang, China; 3 The First Affiliated Hospital, Huzhou University, Huzhou, Zhejiang, China; 4 The First Affiliated Hospital, Zhejiang University School of Medicine, Hangzhou, Zhejiang, China

**Keywords:** COPD, Systemic inflammation, Cytokines, IIPS, COPD assessment test, Biomarkers

## Abstract

**Background:**

Chronic obstructive pulmonary disease (COPD) is increasingly recognized as a systemic inflammatory syndrome in which forced expiratory volume in 1 s expressed as a percentage of predicted value (FEV1% predicted) may dissociate from patient-reported symptom burden. This study evaluated an Integrated Inflammatory Profile Score (IIPS) as a potential biological correlate for a more holistic assessment of stable COPD.

**Methods:**

Serum concentrations of 11 cytokines were measured in 62 patients with stable COPD. The IIPS was derived using automated linear modeling with forward stepwise selection guided by the corrected Akaike information criterion (AICc). Associations of the IIPS with COPD Assessment Test (CAT) scores were compared with those of FEV1% predicted.

**Results:**

The optimized IIPS, comprising interferon-gamma (IFN-γ), interleukin-2 (IL-2), and interleukin-10 (IL-10), was significantly associated with FEV1% predicted (R^2^ = 0.1867, *p* = 0.0005) and outperformed the individual cytokines in discriminating spirometric severity (area under the receiver operating characteristic curve (AUC) = 0.7381). For symptom burden, FEV1% predicted showed greater explanatory power for exertional domains, particularly breathlessness (R^2^ = 0.0832) and activities (R^2^ = 0.1280). By contrast, the IIPS showed numerically stronger trend-level associations for sleep (R^2^ = 0.0519, *p* = 0.0750) and energy (R^2^ = 0.0511, *p* = 0.0773), although these associations did not reach conventional statistical significance.

**Conclusion:**

The IIPS reflects Global Initiative for Chronic Obstructive Lung Disease (GOLD)-defined physiological severity more effectively than any single cytokine measured in this study. Its associations with patient-reported systemic symptoms were modest and exploratory, suggesting that integrated inflammatory profiling may complement spirometry in characterizing COPD burden but requires external and longitudinal validation before clinical application.

## Introduction

Chronic obstructive pulmonary disease (COPD) remains a leading cause of global morbidity and mortality and is characterized by persistent respiratory symptoms and progressive airflow limitation (Barnes & Celli, 2009; Chen et al., 2025; Jo, 2022). Beyond localized airway impairment, COPD is increasingly recognized as a complex systemic syndrome involving chronic low-grade inflammation that contributes to a broad spectrum of extra-pulmonary manifestations and adverse clinical outcomes (Agustí et al., 2003; Barnes, 2016). Traditionally, the forced expiratory volume in 1 s expressed as percentage of predicted value (FEV1% predicted) has served as a core metric for diagnosis, physiological assessment, and severity staging in routine respiratory practice (Bhatt et al., 2023; Mirza et al., 2018). However, accumulating evidence has highlighted a clinically important dissociation between mechanical airflow obstruction and the symptom burden perceived by patients. In many cases, FEV1% predicted does not adequately reflect heterogeneity in exercise limitation, health status impairment, or the overall lived experience of disease (Bajpai et al., 2019; Lange et al., 2015; Lange et al., 2016).

To narrow this gap, patient-reported outcome (PRO) instruments—most notably the COPD Assessment Test (CAT)—have been increasingly adopted to provide a multidimensional evaluation of disease impact in daily life (Gupta et al., 2014; Jones et al., 2009; Miravitlles et al., 2019). The CAT captures symptom burden across several clinically relevant domains, including respiratory discomfort, activity limitation, confidence, sleep, and energy. This multidimensional structure is particularly valuable because it reflects aspects of disease burden that may not be fully represented by spirometric indices alone. At the same time, because CAT scores are inherently subjective and can be influenced by psychological state, comorbidity burden, and inter-individual differences in symptom perception, there remains a clear need for objective biological correlates that can help map measurable pathophysiology onto patient-reported disease experience (Kelly et al., 2012; Miravitlles et al., 2019).

The systemic inflammatory landscape of COPD is characterized by a complex milieu of circulating mediators that participate in persistent airway injury, immune dysregulation, skeletal muscle dysfunction, and multi-organ effects beyond the lung itself (Antoniu, 2010; Gan et al., 2004; King, 2015). A broad range of blood-based inflammatory markers-including acute-phase reactants such as C-reactive protein (CRP) and fibrinogen, as well as cytokines such as interleukin-6 (IL-6) and tumor necrosis factor-α (TNF-α)-has been investigated for its potential to predict exacerbations, lung function decline, and adverse prognosis (Joppa et al., 2006; Mukhopadhyay, Hoidal & Mukherjee, 2006; Pinto-Plata et al., 2012). Nevertheless, efforts to translate single inflammatory biomarkers into routine clinical tools have produced inconsistent results, and associations with symptom burden or disease phenotype have often been modest and unstable across cohorts (Dickens et al., 2011; Garudadri & Woodruff, 2018). This limited performance likely reflects the fact that cytokines do not operate independently; instead, they function within an integrated and partially redundant signaling network, such that any single mediator captures only one fragment of the broader systemic inflammatory state.

Consequently, reliance on individual cytokines provides only a partial and sometimes misleading representation of COPD-related inflammation, making it difficult to derive a universally reliable biomarker from a single molecule. This challenge has been emphasized by studies showing that COPD is biologically heterogeneous, clinically multidimensional, and dynamically shaped by interacting inflammatory pathways rather than by a solitary dominant signal (Agustí et al., 2012; Hlapcic et al., 2020; Li et al., 2025; Pinto-Plata et al., 2007; Singh, Kabra & Goyal, 2026). Accordingly, a more integrated biomarker strategy may be better suited to capturing the holistic systemic burden of COPD than conventional single-marker approaches. Such an integrated framework is also conceptually aligned with the broader movement in biomarker research away from isolated analytes and toward multivariable biological signatures that better reflect complex disease states.

In response to this need, we developed an Integrated Inflammatory Profile Score (IIPS) based on a panel of 11 circulating cytokines measured in patients with stable COPD. Using a statistical framework centered on the corrected Akaike information criterion (AICc), we sought to identify the most informative combination of inflammatory mediators and to determine whether a composite score could better reflect disease severity than any individual cytokine alone. In the final optimized model, IFN-γ, IL-2, and IL-10 were retained as the core contributors to the score. These cytokines represent biologically relevant yet functionally distinct components of immune regulation, encompassing pro-inflammatory signaling, lymphocyte activation, and counter-regulatory anti-inflammatory activity. Their joint inclusion therefore provides a more balanced representation of systemic immune status than a single-marker model would permit. At a practical level, these analytes are also clinically accessible and well established in immunological research, supporting the translational plausibility of a composite inflammatory metric.

The present study was designed not merely to construct a cytokine-derived index, but to examine whether such an index could help bridge objective physiological impairment and multidimensional symptom burden as quantified by the CAT (Wang et al., 2025). We hypothesized that by aggregating synergistic inflammatory information, the IIPS would show a stronger relationship with Global Initiative for Chronic Obstructive Lung Disease (GOLD)-defined physiological severity and might provide complementary explanatory value for symptom domains that are often relatively dissociated from the degree of airflow obstruction, particularly sleep disturbance and energy depletion. This question is clinically relevant because patients with similar spirometric impairment may report markedly different overall disease burdens, suggesting that biological processes beyond airflow limitation contribute meaningfully to how COPD is experienced.

Therefore, the primary objective of this study was to evaluate the performance of the IIPS as an integrated inflammatory correlate in stable COPD. Specifically, we sought to: (1) assess whether the IIPS was associated with GOLD-defined severity across the physiological spectrum of disease; (2) compare its performance with that of individual cytokines in relation to spirometric severity; and (3) examine whether the IIPS showed complementary associations with CAT total score and individual CAT domains. Given the exploratory and cross-sectional nature of the present work, we aimed to determine whether integrated inflammatory profiling could provide a more comprehensive biological mapping of COPD heterogeneity within an established COPD cohort, rather than to claim immediate clinical replacement of spirometry or definitive prediction of patient-reported outcomes.

## Materials and Methods

### Study population and design

This cross-sectional, observational study was designed to investigate the association between serum inflammatory profiles and clinical symptoms in a cohort of patients with stable COPD, with the specific aim of characterizing internal phenotypic heterogeneity and the “clinical dissociation” between lung function and systemic symptom burden beyond traditional spirometric measures. A total of 62 patients with confirmed stable COPD were recruited from the Department of Respiratory and Critical Care Medicine at the First Affiliated Hospital, Huzhou University, between January 1, 2018, and June 30, 2019.

Because the analysis focused on mapping inflammatory trajectories across the established disease severity spectrum (GOLD stages 1–4) within COPD itself, healthy individuals were not included as a control group. The diagnosis and severity of COPD were established according to the GOLD criteria, eligibility required a FEV1/FVC ratio of less than 0.70 (pre- or post-bronchodilator) or a clinical diagnosis of COPD confirmed by a pulmonologist. All participants were required to be in a clinically stable state, defined as no acute exacerbation or significant changes in medication for at least 4 weeks before assessment. Exclusion criteria were applied to minimize confounding systemic inflammatory influences and included other chronic respiratory diseases such as asthma, bronchiectasis, or active pulmonary tuberculosis; acute respiratory infection or COPD exacerbation within the preceding month; coexisting major systemic inflammatory disorders such as rheumatoid arthritis; and significant comorbidities including lung cancer, severe renal failure, or advanced hepatic dysfunction. Notably, all serum samples used for cytokine analysis were obtained from residual blood specimens after routine clinical laboratory testing, an approach adopted to minimize additional invasive procedures while maintaining compatibility with standard clinical workflows. The study protocol was approved by the Human Research Ethics Committee of the First Affiliated Hospital, Huzhou University (IRB approval no. 2017070), and all procedures were conducted in accordance with the Declaration of Helsinki. Written informed consent was obtained from all participants before the collection of clinical data or biological samples.

### Clinical assessment and data collection

Standardized clinical assessments were conducted for all participants during their clinical visit. Pulmonary function tests (PFTs) were performed using an electronic spirometer according to the American Thoracic Society/European Respiratory Society (ATS/ERS) guidelines (Laszlo, 2006). The severity of airflow limitation was classified according to the GOLD guidelines: GOLD-1, FEV1% predicted ≥ 80%; GOLD-2, 50% ≤ FEV1% predicted < 80%; GOLD-3, 30% ≤ FEV1% predicted < 50%; and GOLD-4, FEV1% predicted < 30%. Concurrent with the physiological measurements, the CAT was administered to evaluate the patients’ symptom burden. The CAT consists of eight individual items (cough, phlegm, chest tightness, breathlessness, activities, confidence, sleep, and energy), with each item scored from 0 to 5. The total score (0–40) was calculated as the sum of all items, where higher scores indicate greater disease impact (Guan et al., 2018; Jones et al., 2009). All assessments and blood sampling were performed on the same day to ensure the temporal consistency of the biological and clinical data.

### Serum biomarker analysis

Serum samples were obtained from residual clinical specimens following routine laboratory testing, ensuring no additional invasive procedures for the participants. Following collection, the specimens were immediately aliquoted and stored at −80 °C to ensure cytokine stability. While pre-analytical handling and long-term storage can significantly impact blood cytokine stability (Verberk et al., 2021), our protocol minimized these effects by using immediate aliquoting and strictly limiting the samples to a single freeze-thaw cycle.

Serum cytokine concentrations were quantified using LEGENDplex™ Human (Panel 1) multiplex bead-based immunoassays (BioLegend, San Diego, CA, USA) according to the manufacturer’s instructions, and analyzed using LEGENDplex™ Data Analysis Software (BioLegend, San Diego, CA, USA). The initial panel included: Interleukin (IL)-5, IL-13, IL-2, IL-6, IL-9, IL-10, IFN-γ, TNF-α, IL-17A, IL-17F, IL-4, IL-21, and IL-22. The assays were performed strictly following the manufacturer’s protocol.

In brief, samples were incubated with fluorescence-encoded beads, followed by the addition of detection antibodies and streptavidin-phycoerythrin. Data acquisition was performed on a flow cytometer. Cytokine concentrations (expressed in pg/mL) were determined by referencing standard curves generated concurrently for each assay. Samples were analyzed in singlicate, while calibration standards and quality controls were included in each run to ensure assay performance. This approach was adopted based on the established reproducibility of bead-based multiplex assays and the limited sample volume available. To ensure the statistical robustness of the downstream analysis, cytokines with a detection rate of less than 30% were excluded from the study. Consequently, IL-17A and IL-17F were removed, and the remaining 11 cytokines were included in the final data analysis and model construction.

### Statistical analysis

Data analysis was performed using IBM SPSS Statistics for Windows, Version 24.0 (IBM Corp., Armonk, NY, USA) and GraphPad Prism, Version 10.0 (GraphPad Software, Boston, MA, USA). Continuous variables were expressed as mean ± standard deviation (SD) for normally distributed data or as median (interquartile range, IQR) for non-normally distributed data. A *post-hoc* sex-stratified analysis was also performed to explore whether the associations between the IIPS, FEV1% predicted, and CAT-derived symptom domains differed by sex.

To construct the IIPS, we utilized the Automated Linear Modeling (ALM) framework in SPSS version 24.0, conceptualizing the GOLD staging (grades 1–4) as a continuous spectrum of physiological severity. The Auto Data Preparation (ADP) function was enabled to algorithmically handle outliers and automatically apply optimal transformations to the skewed cytokine data for modeling purposes. A forward stepwise linear regression, guided by minimizing the AICc, was employed to identify the most predictive combination of cytokines. Through this algorithmic process, IFN-γ, IL-2, and IL-10 were selected. The relative contribution of each cytokine to the model was quantified using the Predictor Importance metric (derived from the Type III sum of squares), and the final IIPS for each patient was strictly defined as the standardized predicted value generated by this optimized model.

For downstream validation and correlation analyses, raw cytokine concentrations were logarithmically transformed (*Log*_10_) where appropriate to satisfy normality assumptions. Bivariate associations between individual cytokines, the IIPS, lung function parameters (*FEV_1%* predicted), and CAT scores were evaluated using Pearson or Spearman rank correlation coefficients based on data distribution. The diagnostic performance of the IIPS and individual cytokines in differentiating advanced disease severity (GOLD 3–4 *vs*. GOLD 1–2) was assessed using Receiver Operating Characteristic (ROC) curve analysis, with the calculation of the Area Under the Curve (AUC), sensitivity, and specificity. Simple linear regression models were employed to further quantify the explanatory power of the IIPS and *FEV_1%* predicted for the total CAT score and its eight individual sub-dimensions. A two-sided *p*-value of < 0.05 was considered statistically significant for all analyses.

To facilitate the visualization and exploration of the Predictor Importance weights (IFN-γ, IL-2, and IL-10) and their collective contribution to the IIPS, an interactive web-based application, the ‘IIPS Explorer’, was developed and is accessible at: https://iips-explorer-r9pvww4veorzvyxksrxadn.streamlit.app/.

This tool allows users to simulate patient cytokine profiles and observe real-time score calculations and severity mapping.

## Results

### Clinical characteristics and distribution of serum cytokines

The clinical and demographic characteristics of the 62 patients with stable COPD are summarized in Table 1. The cohort had a mean age of 72.7 ± 8.0 years and was predominantly male (85.5%), reflecting local epidemiological patterns where smoking prevalence is higher among men. The study population encompassed a spectrum of airflow limitation severity (GOLD 1–4), though the distribution was notably skewed toward severe stages, with GOLD 3 and 4 patients accounting for 69.4% of the total cohort. The mean FEV1% predicted was 43.5 ± 22.2%, and the mean total CAT score was 24.5 ± 6.6, indicating a high symptomatic burden.

**Table 1:** Demographic and clinical characteristics of the study population.

Characteristic	COPD cohort (*n* = 62)
**Demographics**	
Age (years), mean ± SD	72.7 ± 8.0
Sex (male), *n* (%)	53 (85.5)
BMI (kg/m^2^), mean ± SD	20.3 ± 3.3
**Lung function**	
FEV1 (% predicted), mean ± SD	43.5 ± 22.2
FEV1/FVC ratio, mean ± SD	0.63 ± 0.16
**Symptom burden**	
CAT score (total), mean ± SD	24.5 ± 6.6
**GOLD stage distribution**, *n* (%)	
GOLD I (mild)	6 (9.7)
GOLD II (moderate)	13 (21.0)
GOLD III (severe)	22 (35.5)
GOLD IV (very severe)	21 (33.9)

**Note:**

BMI, body mass index; CAT, COPD assessment test; COPD, chronic obstructive pulmonary disease; FEV1, forced expiratory volume in 1 s; FVC, forced vital capacity; GOLD, global initiative for chronic obstructive lung disease.

To visualize the systemic inflammatory landscape, we generated a heatmap (Fig. 1A) displaying the 11 serum cytokines (following log10-transformation and z-score normalization) alongside total CAT and sub-dimension scores, sorted by decreasing FEV1% predicted. Visual inspection revealed substantial inter-individual variability in cytokine levels across all GOLD stages. No uniform, monotonic pattern of cytokine expression was observed in relation to the severity of airflow limitation, suggesting that the systemic inflammatory signature in stable COPD is highly heterogeneous and not solely dictated by the degree of mechanical obstruction.

**Figure 1:**
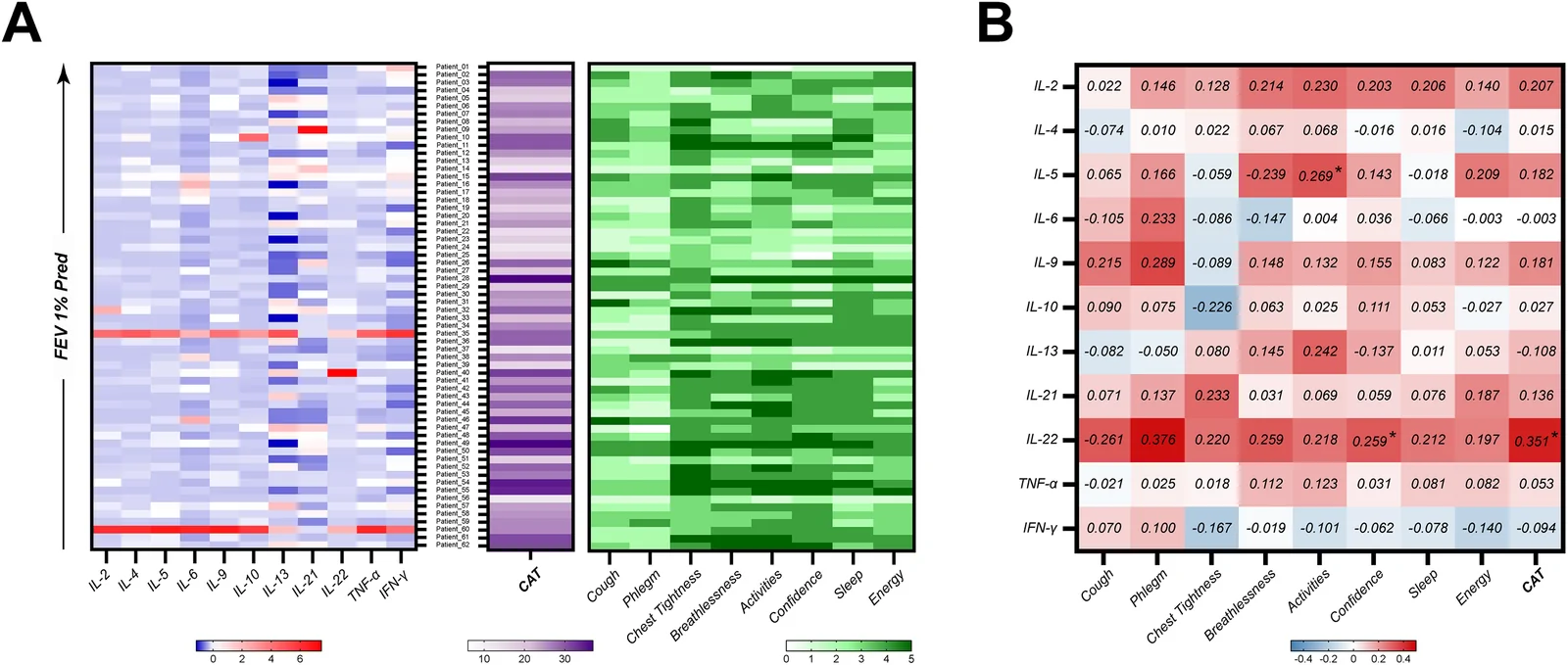
Landscape of serum cytokines and CAT-reported symptoms in stable COPD (*n* = 62). (A) Heatmaps of serum cytokines (left), CAT total score (middle), and CAT item scores (right). Cytokine concentrations were log10-transformed and standardized (z-score) across participants for each biomarker to facilitate comparison. Participants are ordered along the y-axis by airflow limitation based on FEV1% predicted (from lower to higher values, representing severe to mild obstruction). Color bars indicate standardized cytokine levels (blue to red), CAT total score magnitude (light to dark purple), and individual CAT item scores (light to dark green). (B) Spearman correlation matrix relating log10-transformed cytokine concentrations to CAT scores. The matrix displays correlations between cytokines (rows) and the CAT total score as well as eight individual item scores (columns). Cell colors represent the strength and direction of Spearman’s rho (red, positive; blue, negative), with numerical values indicating the correlation coefficients. Asterisks (*) denote nominal statistical significance (*p* < 0.05).

To quantify these relationships, we performed a systematic screening using Spearman rank correlation (Fig. 1B). Several nominal associations were observed, most notably IL-22, which showed nominally positive correlations with Phlegm (rho = 0.376, *p* = 0.0026) and the total CAT score (rho = 0.351, *p* = 0.0052). However, after applying the Benjamini–Hochberg correction to account for the 99 simultaneous correlation pairs (11 cytokines *vs*. CAT total score and eight sub-dimensions), these associations failed to reach the adjusted significance threshold (false discovery rate (FDR)-adjusted *p* < 0.05).

These findings confirm that while certain cytokines may reflect specific symptom dimensions in an exploratory context, no single serum biomarker provides a statistically robust or comprehensive representation of the overall COPD clinical phenotype. Together, these results suggest that single cytokines provide only a partial reflection of the disease’s complexity, supporting the rationale for an integrated index to more effectively characterize the systemic inflammatory landscape.

### Construction and objective validation of the IIPS

To identify an informative inflammatory signature associated with spirometric disease severity, we performed stepwise model selection targeting the GOLD stage using the corrected AICc as the optimization objective (Fig. 2A). IFN-γ was selected in the first step (AICc = −3.441), followed by the addition of IL-2 (Step 2: AICc = −5.590) and IL-10 (Step 3: AICc = −5.619), resulting in progressively lower AICc values and a more parsimonious model. These three cytokines were subsequently integrated, weighted according to the final regression model, to derive the IIPS.

**Figure 2:**
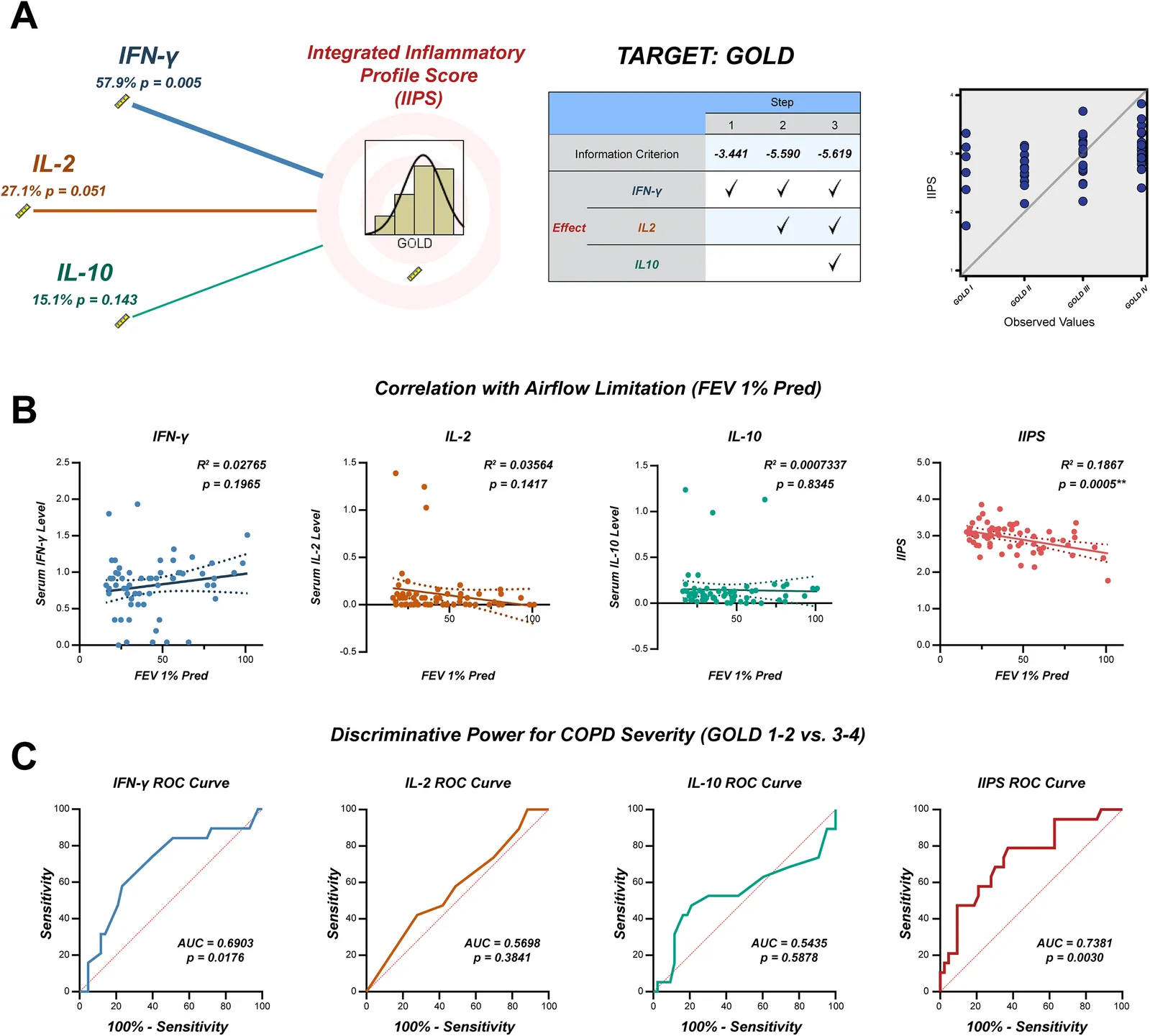
Construction and objective evaluation of the integrated inflammatory profile score (IIPS). (A) Predictor importance of cytokines (IFN-γ, IL-2, and IL-10) selected through the forward stepwise linear regression model (AICc-optimized). The importance weights represent the relative variance explained for the disease severity spectrum (GOLD stages). (B) Linear regression of IFN-γ, IL-2, IL-10, and the composite IIPS against FEV1% predicted. Solid lines indicate fitted regression lines with 95% confidence intervals (dotted lines). R^2^ and *p*-values are reported in each panel. (C) Receiver operating characteristic (ROC) analysis for discriminating GOLD 1–2 *vs*. GOLD 3–4. AUC values and corresponding *p*-values are shown for the IIPS and individual cytokines. Asterisks (**) denote nominal statistical significance (*p* < 0.01).

We next examined the association between the IIPS and airflow limitation using regression analysis with FEV1% predicted (Fig. 2B). Individually, IFN-γ, IL-2, and IL-10 showed weak and non-significant relationships with lung function (IFN-γ: *R*^2^ = 0.02765, *p* = 0.1965; IL-2: *R*^2^ = 0.03564, *p* = 0.1417; IL-10: *R*^2^ = 0.00073, *p* = 0.8345). In contrast, the IIPS exhibited a stronger inverse association with FEV1% predicted (*R*^2^ = 0.1867, *p* = 0.0005), indicating that the integrated inflammatory profile exhibited a stronger association with FEV1% predicted than any single biomarker alone.

Finally, we evaluated the ability of the IIPS to discriminate spirometry severity (GOLD 1–2 *vs*. GOLD 3–4) using ROC analysis (Fig. 2C). The IIPS achieved an AUC of 0.7381 (*p* = 0.0030), exceeding the performance of its individual components: IFN-γ (*AUC* = 0.6903, *p* = 0.0176), IL-2 (*AUC* = 0.5698, *p* = 0.3841), and IL-10 (*AUC* = 0.5435, *p* = 0.5878). At the optimal threshold determined by the Youden index, the IIPS demonstrated an improved balance between sensitivity and specificity compared to single mediators.

Collectively, these findings support the IIPS as an integrative, biologically grounded index associated with GOLD-defined spirometry severity, providing a standardized inflammatory metric that may facilitate objective patient stratification in COPD.

### Bridging utility of IIPS: associations with patient-reported symptom burden

We evaluated the association of the IIPS with patient-reported symptom burden (CAT) and compared its performance with spirometry impairment (FEV1% predicted). In linear regression analyses, FEV1% predicted showed a modest but significant association with the CAT total score (*R*^2^ = 0.0745, *p* = 0.0319), whereas the IIPS demonstrated a borderline positive association (*R*^2^ = 0.0568, *p* = 0.0622) (Fig. 3A). Together, these findings suggest that inflammation-derived and spirometry measures may capture partially distinct components of COPD health status, providing complementary information for comprehensive patient assessment.

**Figure 3:**
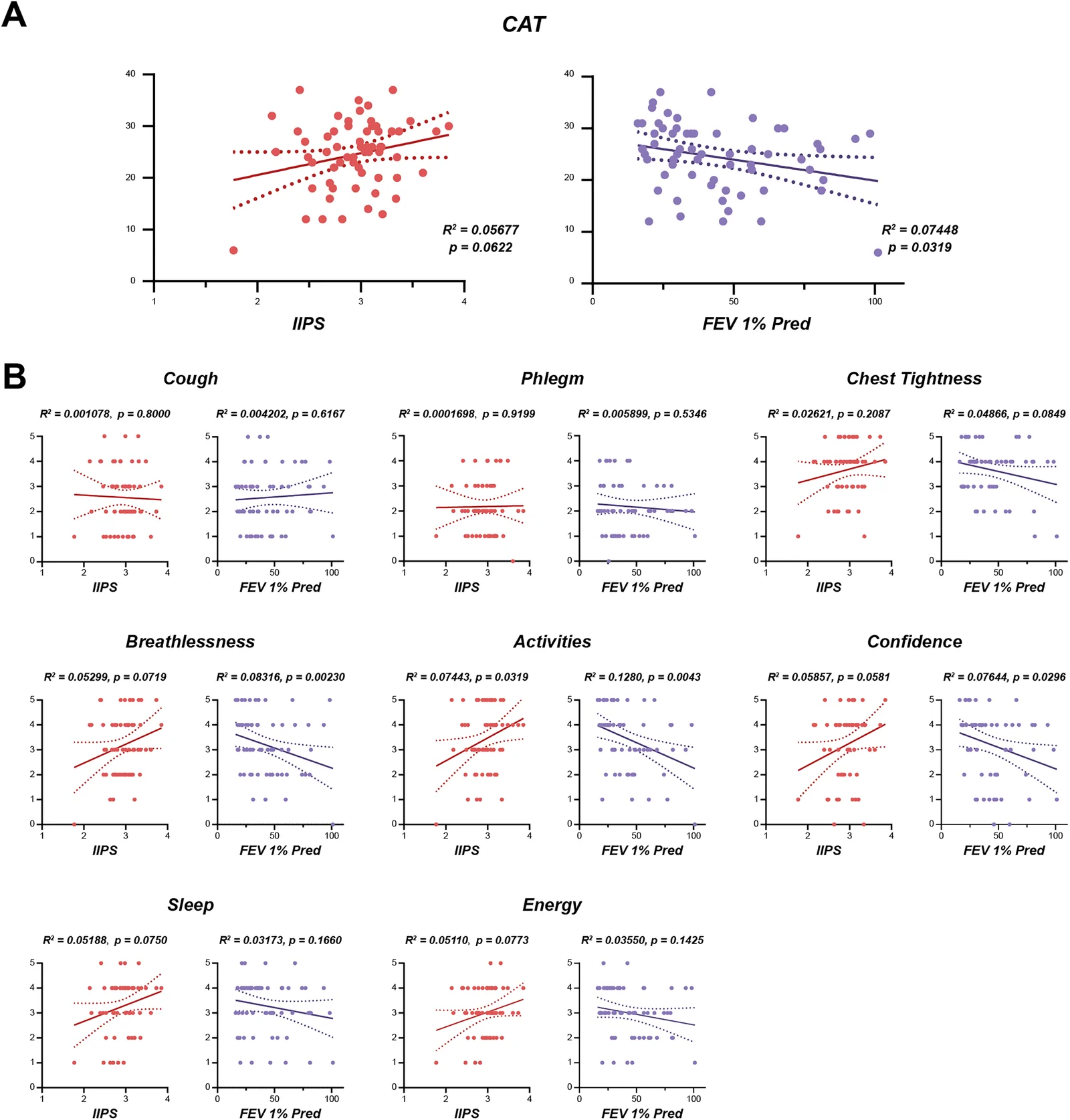
Associations of the IIPS and spirometric impairment with patient-reported symptom burden (*n* = 62). (A) Associations of the CAT total score with objective metrics. Scatter plots illustrate the linear regression relationships between the total COPD assessment test (CAT) score and the integrated inflammatory profile score (IIPS; left, red) or FEV1% predicted (right, purple). R^2^ and *p*-values are reported in each panel. (B) Domain-level regression analyses across eight CAT items. Results are presented as paired panels for each domain, with the IIPS shown on the left (red) and FEV1% predicted on the right (purple). Items are arranged into three thematic rows: Top row (respiratory symptom items): Includes cough, phlegm, and chest tightness, where both metrics exhibited limited explanatory power. Middle row (exertional and functional limitation items): Includes breathlessness, activities, and confidence; FEV1% predicted demonstrated relatively stronger and statistically significant associations in this category. Bottom row (sleep and energy items): includes sleep and energy; the IIPS showed a trend toward higher R^2^ values compared with FEV1% predicted. In all panels, solid lines represent fitted linear regression models, while dotted lines indicate the 95% confidence intervals. Each dot represents an individual participant (*n* = 62). R^2^ and *p*-values for each association are provided within the respective plots.

We next examined domain-level patterns across the eight individual CAT items, following their vertical distribution in Fig. 3B. For local respiratory symptom items located in the upper row (Cough, Phlegm, and Chest Tightness), both metrics exhibited limited explanatory power. Associations for Cough (IIPS: *R*^2^ = 0.0011, *p* = 0.8000; FEV1: *R*^2^ = 0.0042, *p* = 0.6167) and Phlegm (IIPS: *R*^2^ = 0.0002, *p* = 0.9199; FEV1: *R*^2^ = 0.0065, *p* = 0.5346) were weak and non-significant, indicating substantial unexplained variability beyond these two metrics in clinically stable patients.

In contrast, for functional limitation domains in the middle row (Breathlessness, Activities, and Confidence), FEV1% predicted consistently demonstrated stronger and statistically significant associations (Fig. 3B). FEV1% predicted served as a superior predictor for Breathlessness (*R*^2^ = 0.0832, *p* = 0.023), Activities (*R*^2^ = 0.1280, *p* = 0.0043), and Confidence (*R*^2^ = 0.0764, *p* = 0.0296). Notably, while the IIPS was also significantly associated with Activities (*R*^2^ = 0.0744, *p* = 0.0319), the explanatory power of FEV1% predicted remained substantially higher, consistent with the primary role of airflow obstruction in limiting physical function and exertion-related symptoms.

For symptom domains less directly attributable to airflow obstruction, located in the bottom row (Sleep and Energy), the IIPS showed a trend toward relatively stronger associations than FEV1% predicted (Fig. 3B). For these systemic aspects, the IIPS achieved higher R^2^ values and lower *p*-values (Sleep: *R*^2^ = 0.0519, *p* = 0.0750; Energy: *R*^2^ = 0.0511, *p* = 0.0773) compared with FEV1% predicted (Sleep: *R*^2^ = 0.0317, *p* = 0.1660; Energy: *R*^2^ = 0.0355, *p* = 0.1425).

Collectively, these results indicate that while spirometry-related impairment aligns more closely with exertional and functional symptom domains (middle row), the IIPS may better reflect aspects of systemic symptom burden related to sleep and energy (bottom row). This supports the potential bridging role of the IIPS in linking objective systemic inflammation to patient-reported outcomes that are frequently inadequately represented by traditional lung function measures.

A *post-hoc* sex-stratified analysis was performed because the cohort was markedly male-predominant. In the male subgroup (*n* = 53), the IIPS retained a significant association with the Confidence domain (*p* = 0.049), whereas the associations with Sleep and Activities remained non-significant. In the female subgroup (*n* = 9), no statistically robust conclusions could be drawn because of the limited sample size. The full sex-stratified results are provided in Table S2.

## Discussion

The IIPS represents an exploratory biological metric that aligns with physiological severity, although its correlation with subjective symptom burden remained at a trend level and requires further verification. Our initial screening indicated that serum cytokines exhibit weak correlations when considered individually, failing to capture the multifaceted clinical presentation of the disease. By integrating IFN-γ, IL-2, and IL-10, we developed a composite index that not only aligns with traditional spirometry measures but also showed relatively stronger associations, particularly for sleep and energy-related domains. These results support a functional complementarity between clinical metrics: while FEV1% predicted remains more strongly associated with exertional limitations, the IIPS reflects symptom dimensions that are less directly related to the degree of mechanical airflow obstruction.

Previous studies provide important context for the development of integrated biomarker strategies in COPD. Pinto-Plata et al. (2007) used a high-dimensional serum protein platform to screen 143 analytes and identified a 24-biomarker panel associated with FEV1, diffusing capacity, exercise capacity, the BODE index, and exacerbation frequency, providing early proof of concept that multimarker profiles may better represent the heterogeneous clinical expression of COPD than isolated biomarkers. In the longitudinal Evaluation of COPD Longitudinally to Identify Predictive Surrogate Endpoints (ECLIPSE) cohort, Agustí et al. (2012) further demonstrated that persistent systemic inflammation identified a subgroup with increased mortality and exacerbation frequency despite broadly comparable pulmonary abnormalities, indicating that inflammatory heterogeneity may convey information beyond spirometric impairment alone. More recently, a similar systematic review concluded that individual biomarkers frequently show context-dependent performance and that multimarker approaches integrated with clinical parameters may offer greater value for COPD assessment (Singh, Kabra & Goyal, 2026). The present IIPS builds on this rationale using a parsimonious three-cytokine composite to examine physiological severity and symptom-domain heterogeneity within an established COPD cohort; however, its cross-sectional and exploratory design precludes the prognostic interpretation supported by longitudinal studies.

The selection of IFN-γ, IL-2, and IL-10 as the core components of the IIPS highlights the multidimensional nature of systemic inflammation in COPD. IFN-γ emerged as the leading contributor during model selection, which is consistent with evidence implicating Th1-polarized immune responses in chronic airway disease. The inclusion of IL-2 and IL-10 further suggests that the inflammatory burden captured by the IIPS reflects a complex interplay between immune activation, immune regulation, and compensatory anti-inflammatory signaling (Majori et al., 1999; Tang et al., 2023). From a biological perspective, this composite structure may help explain why the IIPS showed a stronger association with physiological severity than the individual cytokines considered separately. However, this interpretation should remain cautious. The current findings support the IIPS as an integrated inflammatory correlate of established COPD severity within this cohort, rather than as evidence of a distinct inflammatory trajectory that can yet be used to infer impending physiological progression.

A central finding of our study is the domain-specific dissociation between spirometric airflow obstruction and patient-reported symptom burden. In our cohort, FEV1% predicted showed greater explanatory power for CAT domains closely linked to respiratory mechanics and physical exertion, particularly Breathlessness, Activities, and Confidence, supporting the established role of spirometry in reflecting functional limitation. By contrast, both FEV1 and the IIPS showed limited explanatory power for the upper-row respiratory symptom items (Cough, Phlegm, and Chest Tightness), suggesting that these manifestations are influenced by additional factors beyond either airflow obstruction or the integrated inflammatory profile in clinically stable disease. Importantly, for symptom domains less directly attributable to mechanical obstruction, the relative pattern shifted: the IIPS showed numerically stronger associations than FEV1% predicted for Sleep and Energy in the full cohort. Although these associations did not reach conventional statistical significance, their directional consistency suggests that integrated inflammatory profiling may capture biological variation related to fatigue, well-being, and other systemic aspects of COPD that are less well represented by spirometry alone. This interpretation is further supported by the sex-stratified analysis, in which the IIPS retained a significant association with the Confidence domain in the male subgroup, pointing to the possibility that inflammatory-symptom coupling may vary across symptom domains and patient subsets. Collectively, these findings do not suggest that the IIPS replaces spirometry; rather, they support the concept that integrated inflammatory signatures and lung function metrics reflect overlapping but non-identical dimensions of COPD burden. In this sense, the IIPS provides a potentially informative biological complement to conventional physiological assessment, particularly for aspects of the patient experience that may be underrepresented by airflow-based measures.

The potential clinical relevance of the IIPS lies in its role as a complementary inflammatory metric alongside conventional spirometry. Although airflow obstruction remains central to COPD severity assessment, it does not fully capture the systemic heterogeneity that may contribute to variable symptom profiles and clinical outcomes. In this setting, an integrated inflammatory score may help describe biological variation that is not fully represented by FEV1% predicted alone. The present findings therefore support the IIPS primarily as an exploratory tool for biological stratification rather than as a clinically actionable marker at this stage. Persistent systemic inflammation has been linked to extra-pulmonary morbidity and impaired quality of life, and future studies may determine whether an index such as the IIPS can improve the characterization of these broader disease features. It is also conceivable that such a score could be evaluated in longitudinal studies or clinical trials as an adjunctive biomarker for monitoring systemic inflammatory status. However, these potential applications remain speculative and will require independent validation, reproducibility testing, and demonstration of added value beyond established clinical measures.

This study has several strengths, primarily the conceptual shift from single-biomarker analysis to an integrative approach through the development of the IIPS. By examining domain-level associations between individual CAT items and biological metrics, we observed patterns suggesting that inflammatory profiling may be more informative for symptom dimensions—such as sleep and energy—that show weaker dependence on mechanical airflow obstruction. This approach provides a complementary objective metric for characterizing the systemic burden of COPD that traditional physiological testing often fails to capture.

Several limitations should be acknowledged. First, the cross-sectional design precludes determination of the temporal relationship between systemic inflammation and symptom burden and does not allow inference regarding whether changes in the IIPS precede shifts in patient-reported outcomes. Second, the cohort was relatively small (*n* = 62), enriched for advanced disease stages (69.4% classified as GOLD 3 or 4), and markedly male-predominant, which together may have limited statistical power, constrained subgroup analyses, and reduced the generalizability of the present findings across the broader COPD population. For this reason, the *post-hoc* sex-stratified analysis should be interpreted cautiously, particularly for the underpowered female subgroup, and the full results are provided in Table S2 for transparency. Third, although the IIPS was derived through an optimized model-selection process, its reproducibility, transportability, and clinically useful thresholds remain to be established in independent external cohorts; in addition, because the current model was optimized against GOLD-defined physiological severity, future studies should determine whether models trained directly on patient-reported outcomes may better capture subjective symptom burden. Finally, the use of long-stored residual clinical samples, despite storage at −80 °C, precluded repeat testing because of specimen exhaustion and leaves open the possibility of long-term protein degradation, while all assays were performed in singlicate because of limited sample volume; although bead-based multiplex platforms provide substantial intra-well signal information, the absence of technical replicates remains an important methodological limitation, and future validation studies should incorporate replicate measurements and assay reproducibility assessment (Ellington et al., 2009).

## Conclusion

In conclusion, this study suggests that the IIPS, derived from IFN-γ, IL-2, and IL-10, may serve as an exploratory biological metric for the multidimensional assessment of stable COPD. Within this cohort, the IIPS showed a stronger relationship with GOLD-defined physiological severity than any individual cytokine alone and revealed a domain-specific pattern in which spirometry remained more closely aligned with exertional and functional limitation, whereas the integrated inflammatory profile showed relatively stronger associations with selected systemic symptom domains, particularly sleep and energy. Although these symptom-related findings remained exploratory and require confirmation, they support the broader concept that inflammatory profiling and lung function testing may capture overlapping but non-identical dimensions of COPD burden. Taken together, the present results highlight the potential value of integrated inflammatory signatures as a complement to conventional physiological assessment while also underscoring the need for validation in larger, prospective, and more balanced cohorts before the IIPS can be considered for robust phenotyping, prognostic evaluation, or treatment-monitoring applications.

## Supplemental Information

10.7717/peerj.21751/supp-1 Supplemental Information 1 STROBE checklist.

10.7717/peerj.21751/supp-2 Supplemental Information 2 Raw data.

10.7717/peerj.21751/supp-3 Supplemental Information 3 Sex-stratified analysis of associations between IIPS, FEV1% predicted, and CAT outcomes. The *post-hoc* sex-stratified results for the total cohort (*n* = 62), male subgroup (*n* = 53), and female subgroup (*n* = 9), including CAT total score and individual CAT domains. Corresponding association statistics for IIPS and FEV1% predicted are shown for each subgroup. Owing to the limited female sample size, subgroup-specific findings should be interpreted with caution.

10.7717/peerj.21751/supp-4 Supplemental Information 4 SPSS Output for IIPS Model.

10.7717/peerj.21751/supp-5 Supplemental Information 5 Codebook for Raw Data.

## Additional Information and Declarations

### Data Availability

The following information was supplied regarding data availability:

The raw measurements are available in the Supplemental Files.
