# Supplementary material for: A serum cytokine-derived integrated inflammatory profile score (IIPS) links systemic inflammation to symptom burden and severity in COPD
Source: PeerJ. 2026 Oct 1;14:e21751. doi: 10.7717/peerj.21751 (PMC13634306; doi:10.7717/peerj.21751)
Supplement: Supplementary file 1 [file peerj-14-21751-s004.pdf]

```
*Automatic Linear Modeling.
LINEAR
 /FIELDS TARGET=GOLD INPUTS=IFNr IL2 IL10 il4 il5 il6 il9 il13 il21 il22 tnfa
 /BUILD_OPTIONS OBJECTIVE=STANDARD USE_AUTO_DATA_PREPARATION=TRUE CONFIDENCE_LEVE
L=95 MODEL_SELECTION=FORWARDSTEPWISE CRITERIA_FORWARD_STEPWISE=AICC REPLICATE_RE
SULTS=TRUE SEED=54752075
 /ENSEMBLES COMBINING_RULE_CONTINUOUS=MEAN COMPONENT_MODELS_N=10
 /SAVE PREDICTED_VALUES(Predicted_01).
```

Automatic Linear Modeling

Notes

Output Created		09-JAN-2026 18:50:03
Comments		
Input	Data	E:\OneDrive\01. Academic\Papers\In Writing\CAT and cytokine in COPD\4th Biomarkers\Figure\Figure 2\Fig 2(Z-scored).sav
	Active Dataset	DataSet1
	Filter	<none>
	Weight	<none>
	Split File	<none>
	N of Rows in Working Data File	66
Syntax		LINEAR /FIELDS TARGET=GOLD INPUTS=IFNr IL2 IL10 il4 il5 il6 il9 il13 il21 il22 tnfa /BUILD_OPTIONS OBJECTIVE=STANDARD USE_AUTO_DATA_PREPARATION=TRUE CONFIDENCE_LEVEL=95 MODEL_SELECTION=FORWARDSTEPWISE CRITERIA_FORWARD_STEPWISE=AICC REPLICATE_RESULTS=TRUE SEED=54752075 /ENSEMBLES COMBINING_RULE_CONTINUOUS=MEAN COMPONENT_MODELS_N=10 /SAVE PREDICTED_VALUES...

Notes

Resources	Processor Time	00:00:00.11
	Elapsed Time	00:00:00.24
Variables Created or Modified	Predicted Value	Predicted_01

Warnings

adp: Supervised merging is not performed because there are no categorical inputs.

Case Processing Summary

	N	Percent
Included	62	93.9%
Excluded	4	6.1%
Total	66	100.0%

Model Summary

**Target**	GOLD
**Automatic Data Preparation**	On
**Model Selection Method**	Forward Stepwise
**Information Criterion**	-5.619

The information criterion is used to compare to models. Models with smaller information criterion values fit better.

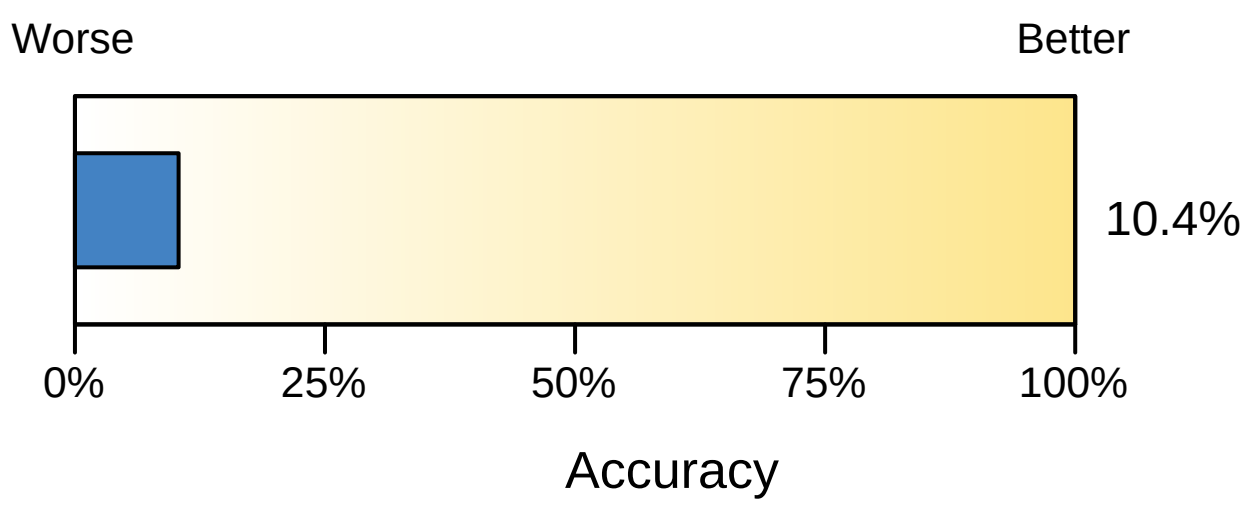

Automatic Data Preparation

Target: GOLD

Field	Role	Actions Taken
**(IFNr_transformed)**	Predictor	Trim outliers
**(IL10_transformed)**	Predictor	Trim outliers<br>Replace missing values
**(il13_transformed)**	Predictor	Trim outliers<br>Replace missing values
**(IL2_transformed)**	Predictor	Trim outliers<br>Replace missing values
**(il21_transformed)**	Predictor	Trim outliers<br>Replace missing values
**(il22_transformed)**	Predictor	Trim outliers<br>Replace missing values
**(il4_transformed)**	Predictor	Trim outliers<br>Replace missing values
**(il5_transformed)**	Predictor	Trim outliers<br>Replace missing values
**(il6_transformed)**	Predictor	Trim outliers<br>Replace missing values
**(il9_transformed)**	Predictor	Trim outliers<br>Replace missing values
**(tnfa_transformed)**	Predictor	Trim outliers<br>Replace missing values

If the original field name is X, then the transformed field is displayed as (X_transformed). The original field is excluded from the analysis and the transformed field is included instead.
One or more records were excluded because of a predictor or target that is missing, a frequency weight that is missing or less than one after rounding, or a regression weight that is missing, negative, or zero.

Predictor Importance

Target: GOLD

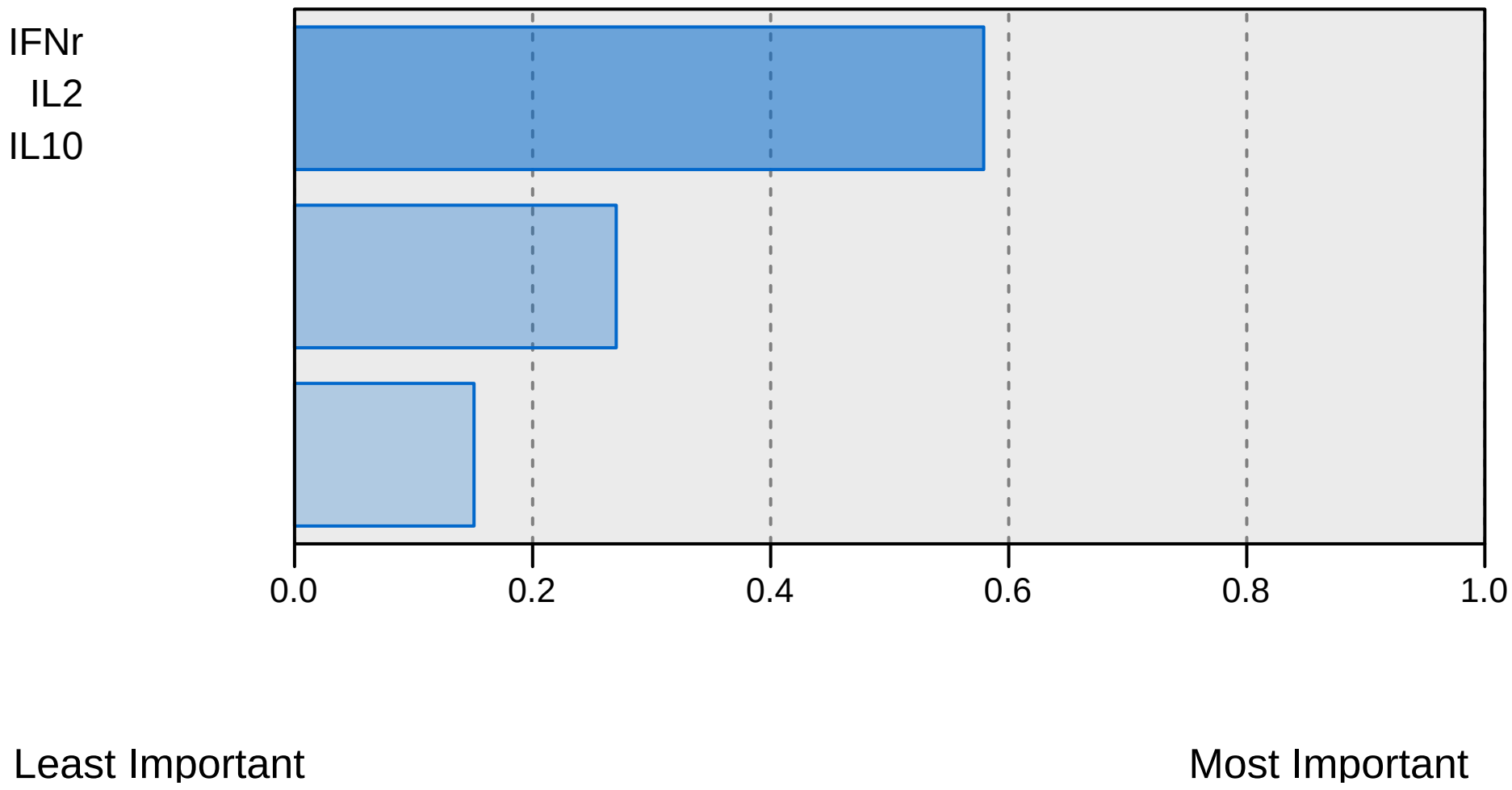

Predicted by Observed

Target: GOLD

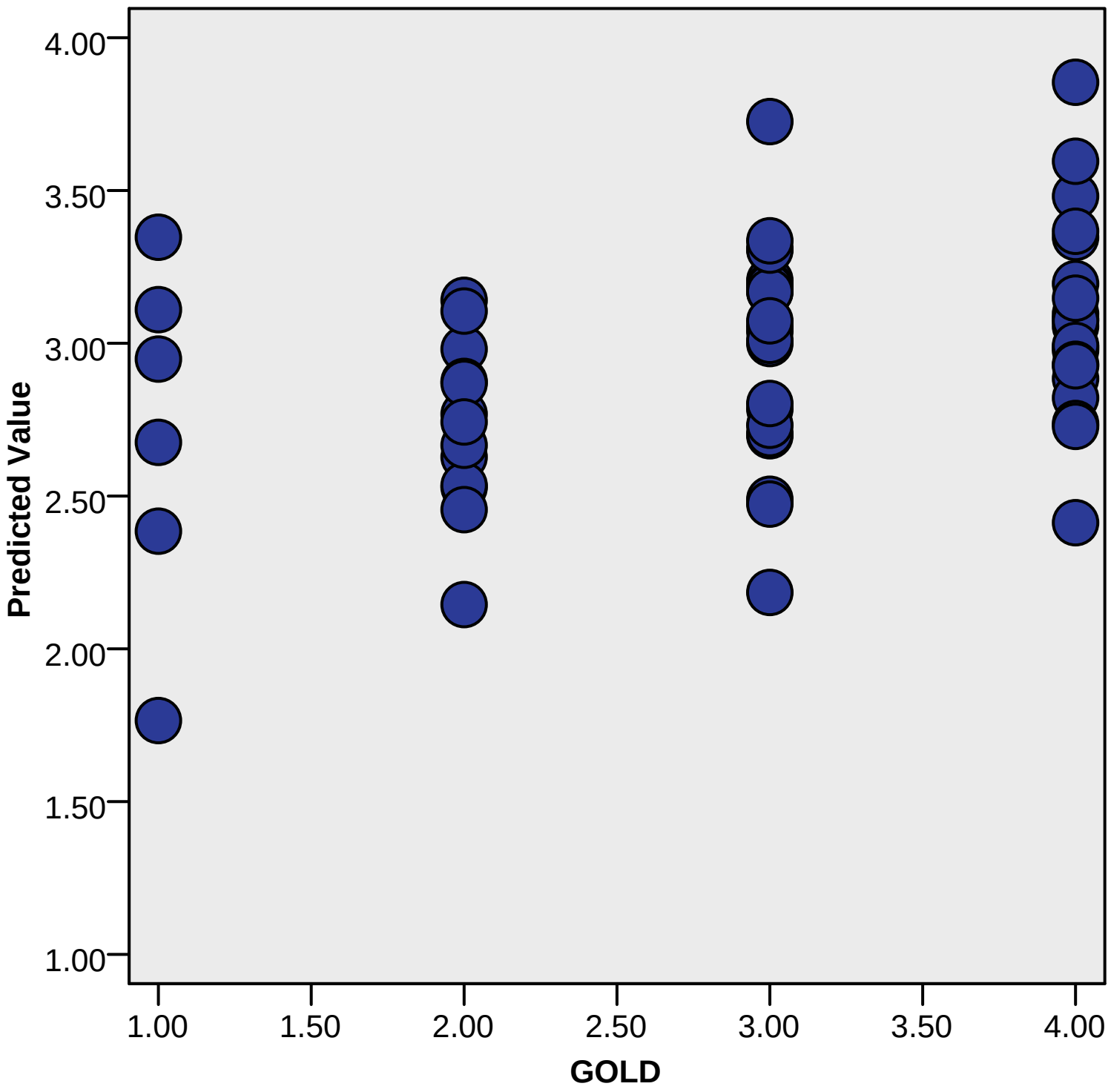

Residuals

Target: GOLD

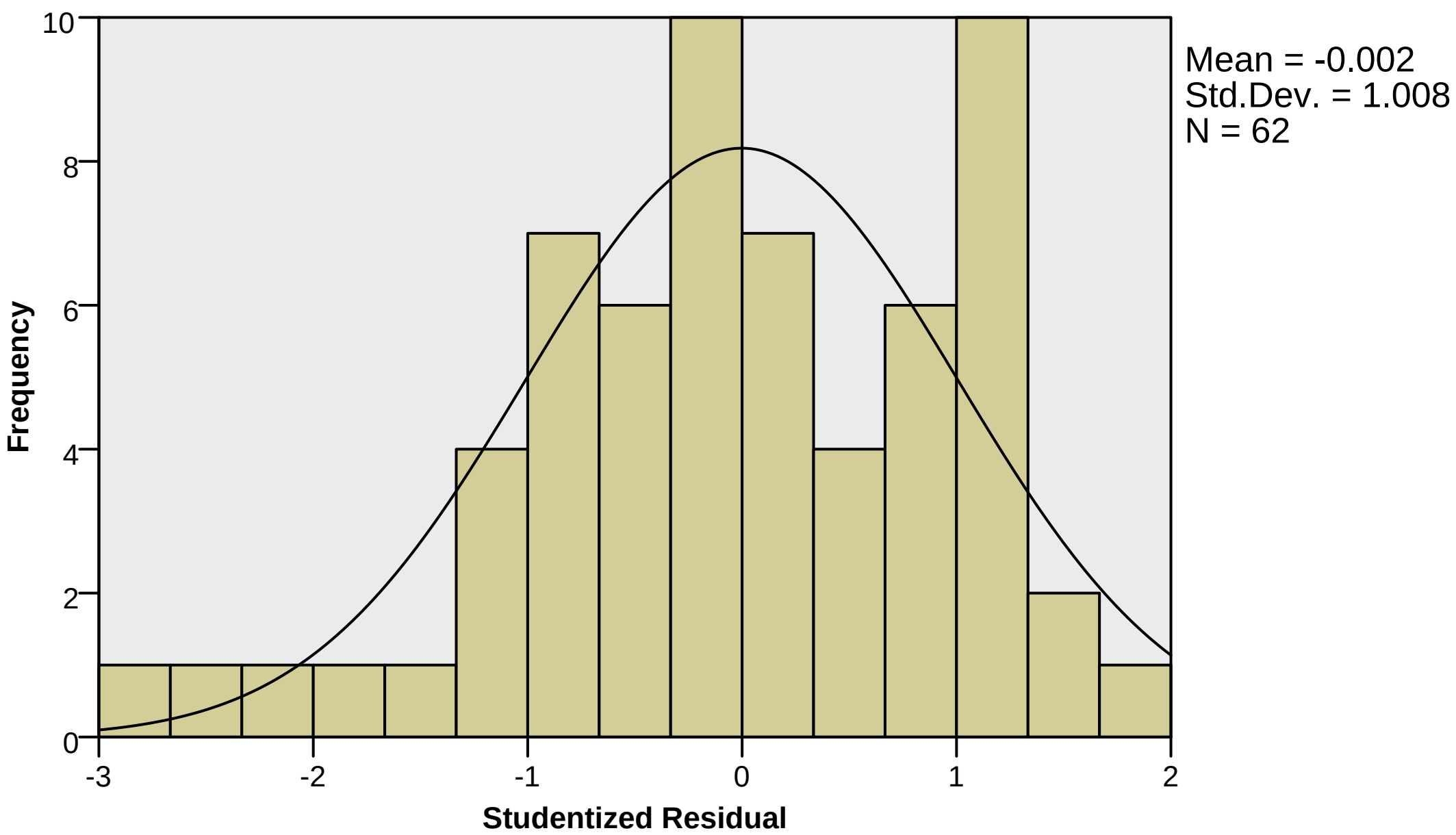

The histogram of Studentized residuals compares the distribution of the residuals to a normal distribution.The smooth line represents the normal distribution.The closer the frequencies of the residuals are to this line, the closer the distribution of the residuals is to the normal distribution.

Residuals

Target: GOLD

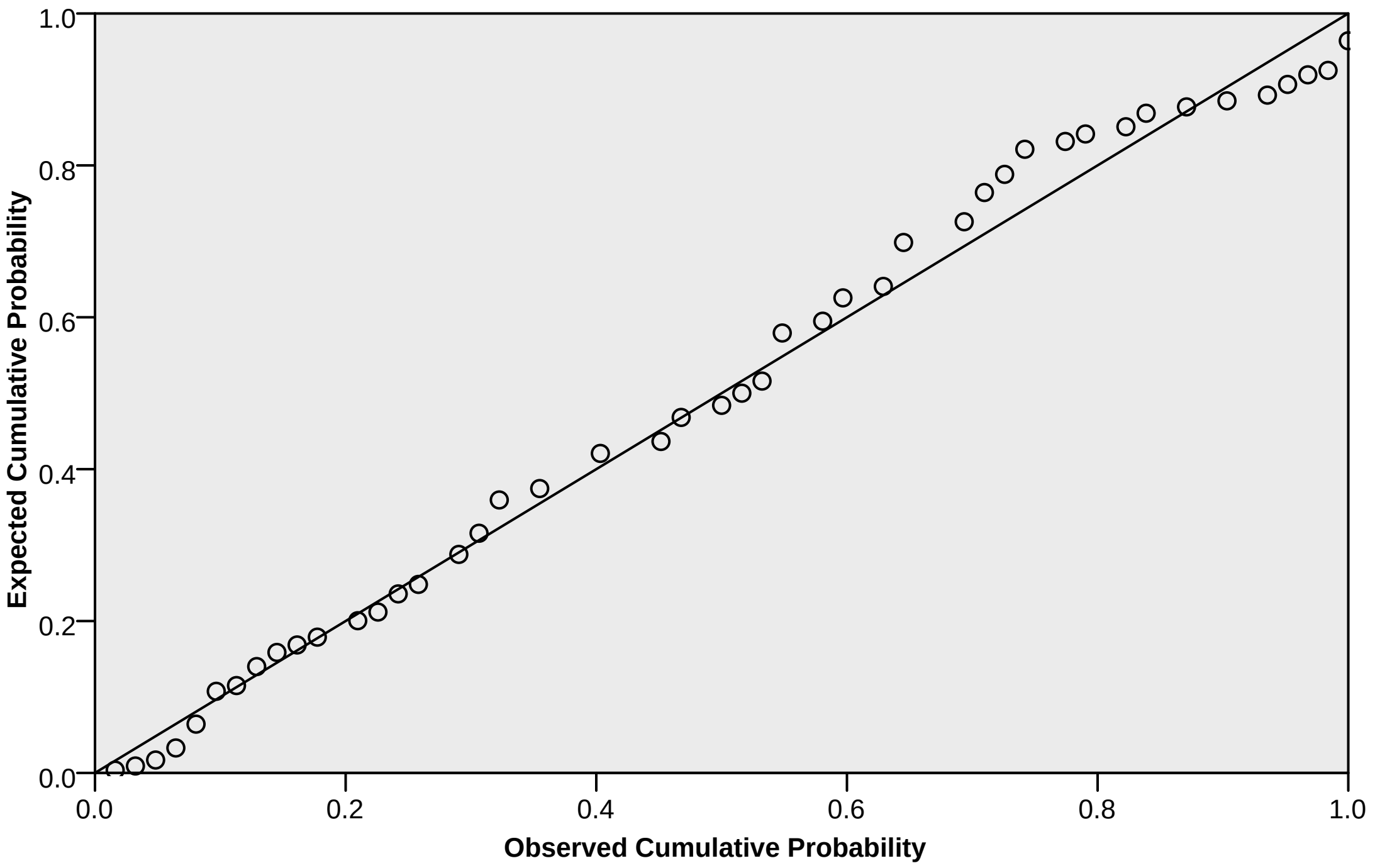

The P-P plot of Studentized residuals compares the distribution of the residuals to a normal distribution.The diagonal line represents the normal distribution.The closer the observed cumulative probabilities of the residuals are to this line, the closer the distribution of the residuals is to the normal distribution.

Outliers

Target: GOLD

Record ID	GOLD	Cook's Distance
**59**	1.00	0.221
**57**	1.00	0.101
**2**	4.00	0.077
**9**	4.00	0.070

Records with large Cook's distance values are highly influential in the model computations. Such records may distort the model accuracy.

Effects

Target: GOLD

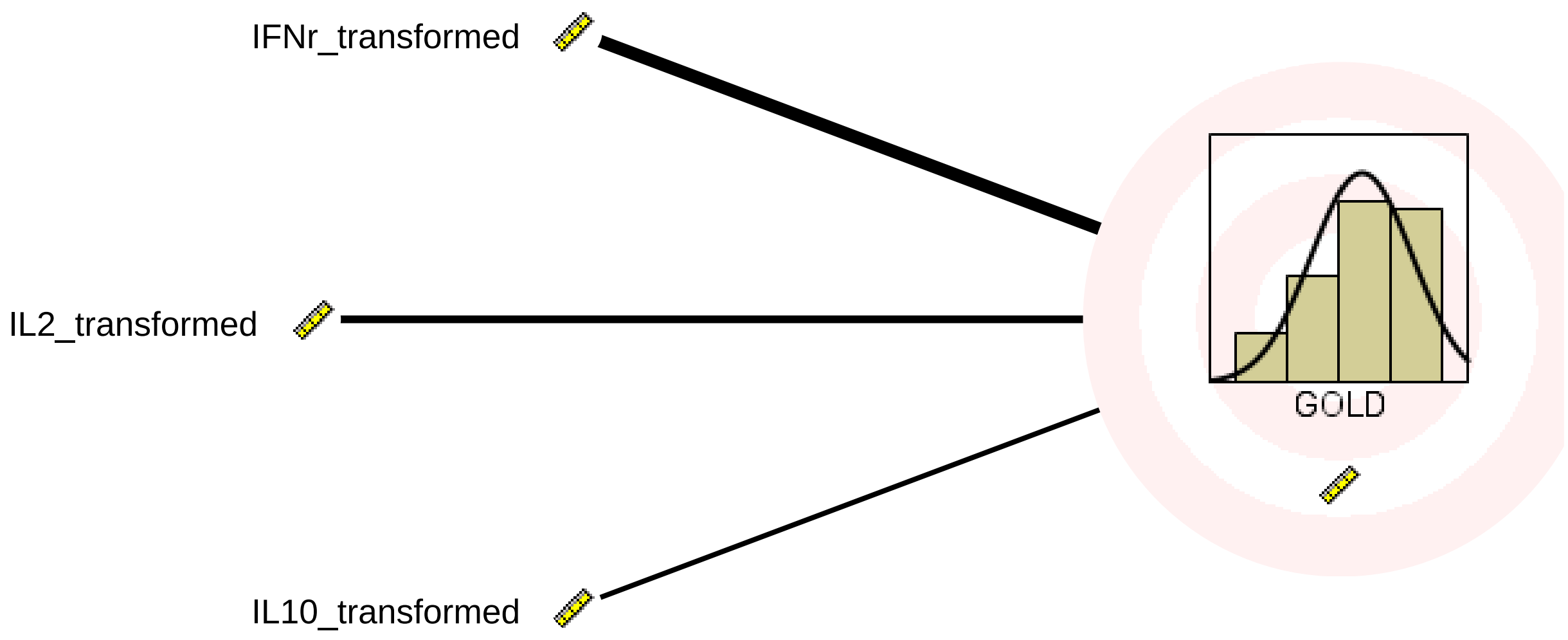

Effects

Target: GOLD

Source	Sum of Squares	df	Mean Square	F	Sig.
Corrected Model	8.529	3	2.843	3.351	.025
Residual	49.213	58	0.848		
Corrected Total	57.742	61			

Effects

Target: GOLD

Source	Sum of Squares	df	Mean Square	F	Sig.	Importance
Corrected Model	8.529	3	2.843	3.351	.025	
IFNr_transformed	7.199	1	7.199	8.484	.005	0.579
IL2_transformed	3.360	1	3.360	3.960	.051	0.270
IL10_transformed	1.874	1	1.874	2.209	.143	0.151
Residual	49.213	58	0.848			
Corrected Total	57.742	61				

Coefficients

Target: GOLD

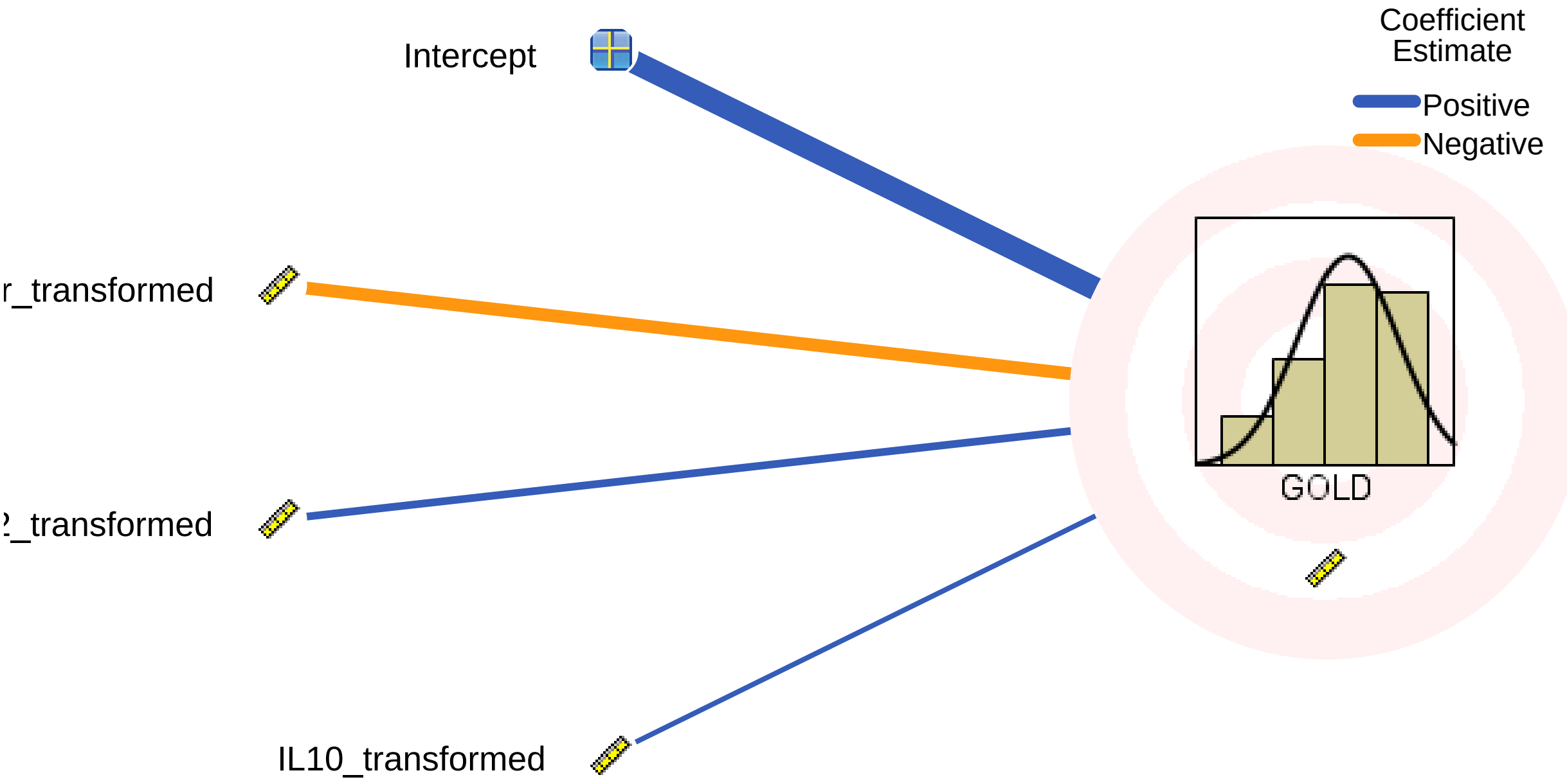

Coefficients

Target: GOLD

Model Term	Coefficient	Sig.	Importance
Intercept	3.115	.000	
IFNr_transformed	-0.091	.005	0.579
IL2_transformed	1.060	.051	0.270
IL10_transformed	0.880	.143	0.151

Coefficients

Target: GOLD

lodel	Term	Coefficient	Std.Error	t	Sig.	95% Confidence Interval		Importanc
						Lower	Upper	
ntercept		3.115	0.209	14.928	.000	2.697	3.532	
FNr_transformed		-0.091	0.031	-2.913	.005	-0.154	-0.029	0.57
.2_transformed		1.060	0.533	1.990	.051	-0.006	2.126	0.27
.10_transformed		0.880	0.592	1.486	.143	-0.305	2.066	0.15

Estimated Means

Target: GOLD

Estimated means charts for the top ten significant effects (p<.05) are …

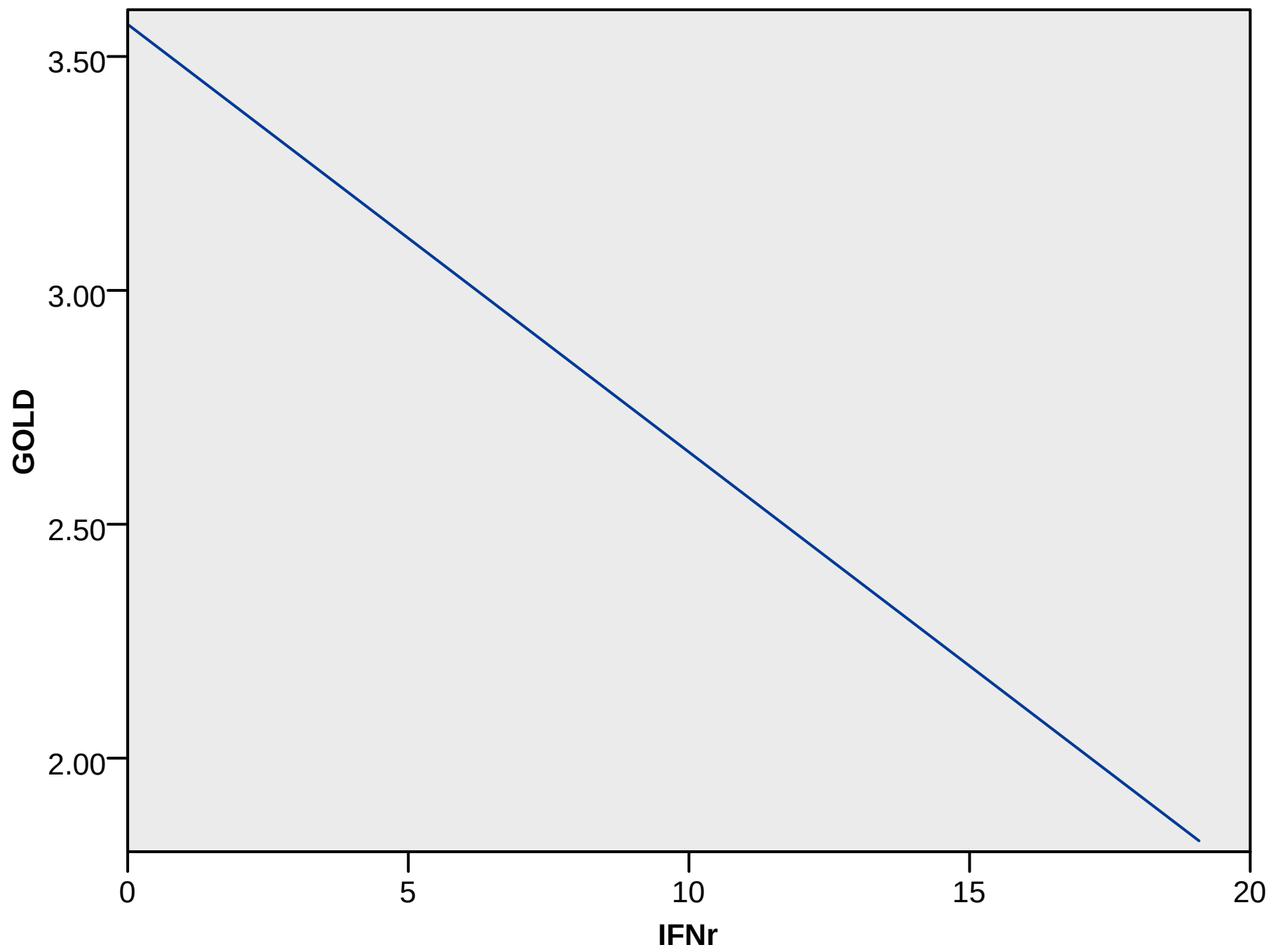

Model Building Summary

Target: GOLD

		Step		
		1	2	3
Information Criterion		-3.441	-5.590	-5.619
Effect	IFNr_transformed	✓	✓	✓
	IL2_transformed		✓	✓
	IL10_transformed			✓

The model building method is Forward Stepwise using the Information Criterion.
A checkmark means the effect is in the model at this step.

Linear Models

Linear models predict a continuous target based on linear relationships between the target and one

- Fields
  - Target
    - GOLD
  - Predictors(Inputs)
  - Use partitioned data: false
- Build Options
- Training Summary
