# Supplementary material for: A serum cytokine-derived integrated inflammatory profile score (IIPS) links systemic inflammation to symptom burden and severity in COPD
Source: PeerJ. 2026 Oct 1;14:e21751. doi: 10.7717/peerj.21751 (PMC13634306; doi:10.7717/peerj.21751)
Supplement: Supplementary file 2 [file peerj-14-21751-s005.docx]

**Study Title:** A Serum Cytokine–Derived Integrated Inflammatory Profile Score (IIPS) Links Systemic Inflammation to Symptom Burden and Severity in COPD.

**Description:** This document provides the variable definitions and encoding for the categorical and continuous data presented in the raw dataset.

**1. Demographic and Anthropometric Variables**

- **Patient**: Unique numeric identifier assigned to each participant to ensure de-identification.
- **Gender**: Categorical variable encoded numerically (1 = Male; 2 = Female).
- **Age**: Continuous variable representing the participant's age in years.
- **Weight (kg)**: Continuous variable representing body weight in kilograms.
- **Height (cm)**: Continuous variable representing height in centimeters.
- **BMI**: Body Mass Index, calculated as weight divided by height squared (kg/m²).

**2. Pulmonary Function Parameters**

- **FEV1 (L)**: Forced Expiratory Volume in 1 second, measured in liters.
- **FVC (L)**: Forced Vital Capacity, measured in liters.
- **FEV1/FVC Ratio**: The ratio of FEV1 to FVC used to establish airflow limitation.
- **FEV1% predicted**: FEV1 expressed as a percentage of the predicted value based on reference standards.

**3. COPD Assessment Test (CAT) Scores**

The CAT is a multidimensional tool used to evaluate the impact of COPD on health status. Each of the eight items is scored on a scale from **0 to 5**, where 0 indicates no impact and 5 indicates maximum impact.

- **Cough (CAT 1)**: Frequency of coughing.
- **Phlegm (CAT 2)**: Presence of mucus in the chest.
- **Chest Tightness (CAT 3)**: Severity of tightness in the chest.
- **Breathlessness (CAT 4)**: Degree of shortness of breath when walking.
- **Activities (CAT 5)**: Level of limitation in home-based activities.
- **Confidence (CAT 6)**: Level of confidence in leaving the home.
- **Sleep (CAT 7)**: Impact of the lung condition on sleep quality.
- **Energy (CAT 8)**: Subjective level of energy or fatigue.
- **CAT Total Score**: The sum of all 8 items (Range: 0–40).

**4. Serum Cytokine Profile**

Numeric concentrations for the following 11 cytokines were quantified using multiplex bead-based immunoassays and are expressed in **pg/mL**:

- **Pro-inflammatory and Immunomodulatory Cytokines**: IL-2, IL-4, IL-5, IL-6, IL-9, IL-10, IL-13, IL-21, IL-22, TNF-alpha, and IFN-gamma.
