# Supplementary material for: A serum cytokine-derived integrated inflammatory profile score (IIPS) links systemic inflammation to symptom burden and severity in COPD
Source: PeerJ. 2026 Oct 1;14:e21751. doi: 10.7717/peerj.21751 (PMC13634306; doi:10.7717/peerj.21751)
Supplement: Supplementary file 3 [file peerj-14-21751-s001.doc]

STROBE Statement—Checklist of items that should be included in reports of ***cross-sectional studies***

|  | Item No | Recommendation | Reported on Page No. / Section |  |  |
| --- | --- | --- | --- | --- | --- |
| **Title and abstract** | 1 | (*a*) Indicate the study’s design with a commonly used term in the title or the abstract | **Page 1 / Title and Abstract** (Manuscript mentions "Integrated Inflammatory Profile Score (IIPS)" and "cross-sectional study"). |  |  |
|  |  | (*b*) Provide in the abstract an informative and balanced summary of what was done and what was found | **Page 1 / Abstract** (Includes Background, Methods, Results, and Conclusion). |  |  |
| Introduction | | |  |  |  |
| Background/rationale | 2 | Explain the scientific background and rationale for the investigation being reported | **Pages 2-3 / Introduction** (Discusses clinical dissociation and the need for a holistic biomarker approach). |  |  |
| Objectives | 3 | State specific objectives, including any prespecified hypotheses | **Page 3 / Introduction** (States the objective to evaluate the IIPS as a novel clinical tool and its hypothesized bridging utility). |  |  |
| Methods | | |  |  |  |
| Study design | 4 | Present key elements of study design early in the paper | **Page 4 / Materials and Methods** (Identified as a cross-sectional, observational study). |  |  |
| Setting | 5 | Describe the setting, locations, and relevant dates, including periods of recruitment, exposure, follow-up, and data collection | **Page 4 / Materials and Methods** (First Affiliated Hospital, Huzhou University, from January 2018 to June 2019). |  |  |
| Participants | 6 | (*a*) Give the eligibility criteria, and the sources and methods of selection of participants | **Page 4 / Materials and Methods** (Lists GOLD criteria for diagnosis and specific exclusion criteria) |  |  |
| Variables | 7 | Clearly define all outcomes, exposures, predictors, potential confounders, and effect modifiers. Give diagnostic criteria, if applicable | **Pages 4-5 / Materials and Methods** (Defines IIPS, FEV1 percent predicted, and CAT scores). |  |  |
| Data sources/ measurement | 8* | For each variable of interest, give sources of data and details of methods of assessment (measurement). Describe comparability of assessment methods if there is more than one group | **Pages 4-5 / Materials and Methods** (Electronic spirometry, CAT scale, and multiplex bead-based immunoassays). |  |  |
| Bias | 9 | Describe any efforts to address potential sources of bias | **Page 4 / Materials and Methods** (Strict exclusion criteria used to minimize confounding systemic inflammatory influences). |  |  |
| Study size | 10 | Explain how the study size was arrived at | **Page 4 / Materials and Methods** (n = 62) and **Page 11 / Discussion** (Acknowledged as a relatively small cohort). |  |  |
| Quantitative variables | 11 | Explain how quantitative variables were handled in the analyses. If applicable, describe which groupings were chosen and why | **Page 5 / Statistical Analysis** (Use of log10 transformation and z-score normalization). |  |  |
| Statistical methods | 12 | (*a*) Describe all statistical methods, including those used to control for confounding | **Page 5 / Statistical Analysis** (AICc-based stepwise selection, correlation analyses, and linear regression). |  |  |
|  |  | (*b*) Describe any methods used to examine subgroups and interactions | **Page 5 / Statistical Analysis** (Differentiation between GOLD 1-2 and GOLD 3-4 stages). |  |  |
|  |  | (*c*) Explain how missing data were addressed | **Page 5 / Serum Biomarker Analysis** (Cytokines with detection rate under 30 percent were excluded). |  |  |
|  |  | (*d*) If applicable, describe analytical methods taking account of sampling strategy | N/A |  |  |
|  |  | (*e*) Describe any sensitivity analyses | N/A |  |  |
| Results | | |  |  |  |
| Participants | 13* | (a) Report numbers of individuals at each stage of study—eg numbers potentially eligible, examined for eligibility, confirmed eligible, included in the study, completing follow-up, and analysed | **Page 6 / Results** (A total of 62 patients included in the final analysis). |  |  |
|  |  | (b) Give reasons for non-participation at each stage | N/A |  |  |
|  |  | (c) Consider use of a flow diagram | N/A |  |  |
| Descriptive data | 14* | (a) Give characteristics of study participants (eg demographic, clinical, social) and information on exposures and potential confounders | **Page 12 / Table 1** (Summarizes age, gender, BMI, smoking history, and lung function). |  |  |
|  |  | (b) Indicate number of participants with missing data for each variable of interest | **Page 6 / Results** (No missing data reported for the final cohort of 62). |  |  |
| Outcome data | 15* | Report numbers of outcome events or summary measures | **Pages 6-9 / Results** (Reports IIPS values, CAT scores, and lung function parameters). |  |  |
| Main results | 16 | (*a*) Give unadjusted estimates and, if applicable, confounder-adjusted estimates and their precision (eg, 95% confidence interval). Make clear which confounders were adjusted for and why they were included | **Pages 7-9 / Results** (Reports R-squared values, p-values, and AUC results). |  |  |
|  |  | (*b*) Report category boundaries when continuous variables were categorized | **Page 4 / Materials and Methods** (GOLD guidelines used for severity classification). |  |  |
|  |  | (*c*) If relevant, consider translating estimates of relative risk into absolute risk for a meaningful time period | N/A |  |  |
| Other analyses | 17 | Report other analyses done—eg analyses of subgroups and interactions, and sensitivity analyses | **Page 6 / Results** (Spearman rank correlation screening for 11 cytokines) |  |  |
| Discussion | | |  |  |  |
| Key results | 18 | Summarise key results with reference to study objectives | **Page 10 / Discussion** (Summarizes the IIPS as a bridge between physiological impairment and symptom burden). |  |  |
| Limitations | 19 | Discuss limitations of the study, taking into account sources of potential bias or imprecision. Discuss both direction and magnitude of any potential bias | **Page 11 / Discussion** (Discusses sample size, severity distribution, and study design). |  |  |
| Interpretation | 20 | Give a cautious overall interpretation of results considering objectives, limitations, multiplicity of analyses, results from similar studies, and other relevant evidence | **Pages 10-12 / Discussion** (Interprets findings regarding functional decoupling and clinical value). |  |  |
| Generalisability | 21 | Discuss the generalisability (external validity) of the study results | **Page 11 / Discussion** (Discusses severity distribution and potential limits on generalizability). |  |  |
| Other information | | |  |  |  |
| Funding | 22 | Give the source of funding and the role of the funders for the present study and, if applicable, for the original study on which the present article is based | **Page 12 / Funding** (National Natural Science Foundation of China, etc.). |  |  |

*Give information separately for exposed and unexposed groups.

**Note:** An Explanation and Elaboration article discusses each checklist item and gives methodological background and published examples of transparent reporting. The STROBE checklist is best used in conjunction with this article (freely available on the Web sites of PLoS Medicine at http://www.plosmedicine.org/, Annals of Internal Medicine at http://www.annals.org/, and Epidemiology at http://www.epidem.com/). Information on the STROBE Initiative is available at www.strobe-statement.org.
